# Association between the Effects of High Temperature on Fertility and Sleep in Female Intra-Specific Hybrids of *Drosophila melanogaster*

**DOI:** 10.3390/insects12040336

**Published:** 2021-04-09

**Authors:** Lyudmila P. Zakharenko, Dmitriy V. Petrovskii, Nataliya V. Dorogova, Arcady A. Putilov

**Affiliations:** 1 Department of Insect Genetics, Institute of Cytology and Genetics of the Siberian Branch, The Russian Academy of Sciences, 630090 Novosibirsk, Russia; zakharlp@bionet.nsc.ru (L.P.Z.); dm_petr@ngs.ru (D.V.P.); dorogova@bionet.nsc.ru (N.V.D.); 2 Faculty of Natural Science, Novosibirsk State University, 630090 Novosibirsk, Russia; 3 Laboratory of Sleep/Wake Neurobiology, The Institute of Higher Nervous Activity and Neurophysiology of the Russian Academy of Sciences, 119991 Moscow, Russia

**Keywords:** fruit fly model, sleep disorders, infertility, reproductive health

## Abstract

**Simple Summary:**

Given the complexity of the human reproduction system and the numerous limitations imposed on human studies, a fruit fly model was used to investigate whether the effects of high temperature on fertility and sleep correlate one with another. No evidence was provided for a causal link of temperature-sensitive sterility with sleep disturbance under high temperature in infertile females born from females of Canton-S strain and males of Harwich strain. However, sensitivity of sleep of these females to high temperature differed from that in fertile females born from females of Harwich strain and males of Canton-S strain and in males of either cross.

**Abstract:**

Humans and fruit flies demonstrate similarity in sleep-wake behavior, e.g., in the pattern of sleep disturbances caused by an exposure to high temperature. Although research has provided evidence for a clear connection between sleeping problems and infertility in women, very little is known regarding the mechanisms underlying this connection. Studies of dysgenic crosses of fruit flies revealed that an exposure to elevated temperature induces sterility in female intra-specific hybrids exclusively in one of two cross directions (progeny of Canton-S females crossed with Harwich males). Given the complexity and limitations of human studies, this fruit flies’ model of temperature-sensitive sterility might be used for testing whether the effects of high temperature on fertility and on 24-h sleep pattern are inter-related. To document this pattern, 315 hybrids were kept for at least five days in constant darkness at 20 °C and 29 °C. No evidence was found for a causal link between sterility and sleep disturbance. However, a diminished thermal responsiveness of sleep was shown by females with temperature-induced sterility, while significant responses to high temperature were still observed in fertile females obtained by crossing in the opposite direction (i.e., Canton-S males with Harwich females) and in fertile males from either cross.

## 1. Introduction

Adequate sleep is crucial for health and well-being. Getting a good sleep helps in refreshing and restoring the immune and physiological systems and in regulating hormonal levels including the levels of fertility-related hormones. Therefore, it comes as no surprise that a large number of diseases demonstrated the associations with the circadian and sleep disruptions. Recent research provided evidence for an important role of circadian rhythms and sleep in the reproductive health of women [1]. Fertility was found to be threatened by the disruptions of the circadian timing system and sleep [2,3,4], and similar adverse effects were found in experiments with animals. For instance, the disruptions of the circadian timing system and sleep-wake behavior resulted in diminished reproductive capacity [5]. By using several animal models, the circadian control of critical reproductive events has been demonstrated at the level of the whole organism [6] and peripheral ovarian clocks [7]. It has also been shown that the neuronal control of the reproductive axis and sleep-generating neurons share an anatomical location [8].

Very little is known regarding the mechanisms underlying the link between fertility and disturbed women’s sleep [9]. Sleep and circadian rhythms might influence fertility in multiple pathways, and it is not excluded that a disturbed or inadequate sleep might produce biological changes resulting in infertility. However, the study data to date do not substantiate any of the proposed pathways (see [9,10] for review). From a human health perspective, a particularly relevant question is why does infertility demonstrate such an inter-relationship with circadian and sleep disturbances? This inter-relationship might be rather complex and, therefore, it has not been well established so far. For instance, complicating the issue is a question of whether circadian and sleep disturbances are the results of infertility, causes of infertility, or whether the relationship between them is one of reciprocal nature [10]. This makes the therapeutic targeting both female reproductive function and sleep disorders difficult. To answering the question of plausibility of causal relationship, it appears to be essential to understand genetic and environmental causes of association of infertility with disruptions of the fundamental circadian and sleep regulation mechanisms.

Given the complexity and limitations of human studies, the fruit fly, *Drosophila melanogaster*, is an ideal model organism for dissecting relationships between genes, environment, sleep-wake behavior, and various diseases [11]. It might, in particular, provide a powerful and rapid platform to study the mechanisms underpinnings the associations between environmental stress, infertility, and sleep-wake disturbances. Hot ambient temperature belongs to the most powerful natural factors causing profound disturbances in the human sleep-wake cycle [12,13]. For instance, a strong annual rhythmicity of sleeping problems has been reported by both newcomers and native residents of a region with hot summer temperature. Even people exposed at high latitudes to very cold winter temperatures and extremely variable photoperiod reported less profound seasonality [14]. In the absence of air conditioning, summer season was found to be associated with a drastic increase of any of several studied symptoms of sleep disturbance, including daytime sleepiness, premature awakening, and difficulties falling and staying asleep [15].

Since sleep pattern in *Drosophila* is very sensitive to heat, it is enticing to draw parallels to sleep-wake behavior of humans exposed to thermal stress [16]. The experimental research indicates that fly’s sleep-wake pattern might be reorganized by an increase of ambient temperature in a way that is very similar to the response of human sleep-wake pattern to heat. For instance, usually nighttime sleep and daytime activity decrease, whereas daytime sleep and early night activity increase due to exposure to high temperature [16,17,18,19].

Additionally, *Drosophila melanogaster* can serve as a model of temperature-sensitive agametic sterility [20], because gonadal dysgenesis in intra-specific hybrids can occur exclusively in one of two cross directions and only due to cultivation under elevated temperature (see also [21,22,23]). Therefore, the effects of high temperature on sleep in sterile F1 female progeny might be compared with the effects on sleep in the reciprocal (fertile) female progeny. Consequently, our major purpose was to try to use this fruit fly model of temperature-sensitive infertility for delineation of causal link between the effects of elevated air temperature on fertility and sleep pattern.

In the present study, we used this fruit flies’ model of temperature-sensitive sterility to compare the responsiveness of the 24-h sleep-wake pattern to such environmental factor as high temperature in genetically identical females (crosses in two directions) that do not demonstrate a difference in reproductive health under normal thermal condition, but demonstrate it under high developmental temperature. Our expectation was to find that the sterile F1 female progeny is associated with the most drastic disturbance of the 24-h sleep pattern under exposure to high temperature. If such an association is documented, further research might be aimed at the elaboration of the underlying mechanisms (i.e., either one of the effects causes another, or they are inter-related due to their links to a 3rd unknown cause).

## 2. Materials and Methods

To create hybrid females characterized by intra-specific paternal-maternal hybrid gonadal dysgenesis, females of Canton-S (♀C-S) strain were crossed with males of Harwich (♂H) strain (♀C-S♂H). The reciprocal (fertile) female hybrids (♀H♂C-S) and fertile hybrid males (♀H♂C-S and ♀C-S♂H) served as three control crosses. F1 hybrids were obtained by breeding 10 females and 10 males in each tube, and these hybrids were maintained at either 20 °C or 29 °C. F70 hybrids of both cross directions were obtained by crossing ♀H♂C-S and ♀C-S♂H at 25 °C (i.e., this temperature does not cause sterility in F1-F70 female progeny). All flies originated from the collection of the Institute of Cytology and Genetics (Novosibirsk) and were cultivated in the laboratory of Department of Insect Genetics of Drosophila. Canton-S and Harwich strains were received in 1996 and 1990 from L.Z. Kaidanov and the Obninsk Drosophila collection, respectively.

To confirm that infertility occurs in females of just one of two crosses under one of two temperature conditions, ovaries of female hybrids (10 for each cross in each temperature condition) were fixed in formaldehyde—3.7% in PBS (Phosphate Buffer, pH 7.2–7.6), and stained with ProLong Gold anti-fade reagent with DAPI (4 ‘, 6-diamidino-2-phenylindole, Molecular Probes/Invitrogen). Images for histological results were obtained using an AxioImager Z1 microscope with ApoTome attachment (Zeiss) and the AxioCam MR and AxioVision software (Zeiss, Germany).

To test survival time under high temperature, males and females from parental strains and their offspring of two cross directions were compared twice, with either F1 or F70. In each of 100 tubes, 20 flies were kept at 29 °C, and the food vials were replaced twice a week after removing and counting dead flies. In total, 2000 flies were counted for survival analysis.

To document 24-h sleep pattern, flies were kept in the laboratory at 25 °C and standard photoperiod (light between 7:00 and 19:00) to age 5–10 days, and then placed individually in glass locomotor-monitoring tubes with standard corn meal media. For evaluation of the effects of high temperature on the 24-h sleep pattern, the measurements were obtained simultaneously for two crosses during, at least, 5 days in constant darkness at either 20 °C or 29 °C. Locomotor activity was monitored in 1-min bins as the numbers of beam breaks using the Drosophila Activity Monitoring System (“Trikinetics”, Waltham, MA, USA). These data on locomotor activity (expressed as the number of beam breaks per each 1-min interval) were used to quantify sleep events defined, in accord with the Donelson et al. [24] criterion, as 5 consecutive minutes of absence of any locomotor activity. Among several other variables, amount of sleep was calculated using data acquisition software package downloaded from the TriKinetics web site (www.trikinetics.com (accessed on 9 April 2021)). The data were transferred from the original software into Excel software for the purpose of further analysis and illustration of results.

To perform statistical analyses, data for the first day were excluded as mainly reflecting the habituation process, whereas the following 30-min estimates of sleep were averaged over four 24-h intervals to obtain measurements of sleep (in min) for each of 48 time points constituting the 24-h cycle of an individual fly. To draw illustrations and to analyze the 24-h sleep pattern, mean values and Standard Error of Mean (SEM) were calculated using the SPSS_22.0_ statistical software package (IBM, Armonk, NY, USA). Two-, three- and four-way repeated measure ANOVAs (rANOVAs) were run on individual 24-h sleep patterns with the repeated measure “Time” (48 time points constituting the 24-h cycle) and one, two and/or three independent factors, “Temperature” (20 °C vs. 29 °C), “Cross direction” (♀H♂C-S vs. ♀C-S♂H), and/or “Sex” (Male vs. Female). Additionally, Student’s test was applied to compare the effects of high temperature on amounts of sleep on 4 6-h time intervals of the 24-h cycle. To evaluate survival rate of hybrid flies, the Kaplan–Meier estimator was used, and Kaplan–Meier curves were compared with the log-rank (Mantel–Cox) test.

## 3. Results

**Figure 1:**
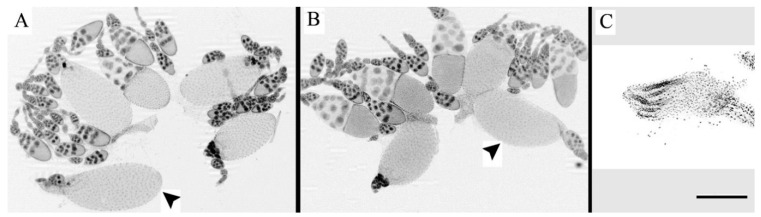
Ovarian morphology of F1 females. (**A**) Ovaries of ♀H♂C-S (non-dysgenic) females after incubation at 29 °C. Ovary contains eggs chambers at different stages of oogenesis and eggs (arrowhead). (**B**) Ovaries of ♀C-S♂H (dysgenic) females at 20 °C. They also have normal morphology. (**C**) Ovaries of ♀C-S♂H (dysgenic) females at 29 °C. Oogenesis does not occur and eggs do not develop. Example of one of 25 flies per each cross and each temperature. Nuclei are stained by DAPI. Scale bar: 12 µm.

As we expected from the previous reports of intra-specific hybrids of fruit flies [20,21,22,23,25], the symptoms of paternal-maternal hybrid gonadal dysgenesis were found only in females of one of two cross directions: when females of Canton-S strain were hybridized with males from Harwich strain (♀C-S♂H). As illustrated in Figure 1, developmental temperature had the critical effect on the manifestation of hybrid dysgenesis. The deleterious effect was found only when eggs were hatched, larvae were reared, and newly emerged flies were permanently kept at 29 °C. As we also expected, sterility of F1 female progeny under high temperature was caused by underdevelopment of ovaries.

The results on survival of parental strains and their F1 and F70 hybrids revealed significantly higher survival rate for infertile and fertile female hybrids compared to male hybrids and parental strains (Table 1). For instance, log rank (Mantel-Cox) test yielded significant difference between F1 female hybrids and either female parents (*χ*^2^ = 186.6, df = 1, *p* < 0.001) or F1 male hybrids (*χ*^2^ = 59.2, df = 1, *p* < 0.001). A higher survival rate of female hybrids persisted over generation (Table 1, right).

Our expectation about the difference between crosses in 24-h sleep pattern was supported by the results of rANOVAs (Table 2). They yielded the significant interactions of repeated measure “Time” with “Cross direction” (Table 2). A triple interaction (“Time” by “Cross direction” by “Sex”) was also significant (Table 2, lower part). Separate rANOVAs of sleep pattern in male and female hybrids suggested a significant interaction between “Time” and “Cross direction” in males (F47/5875 = 6.601, *p* < 0.001), while this interaction was non-significant in females (F47/6392 = 1.073, *p* > 0.05).

**Figure 2:**
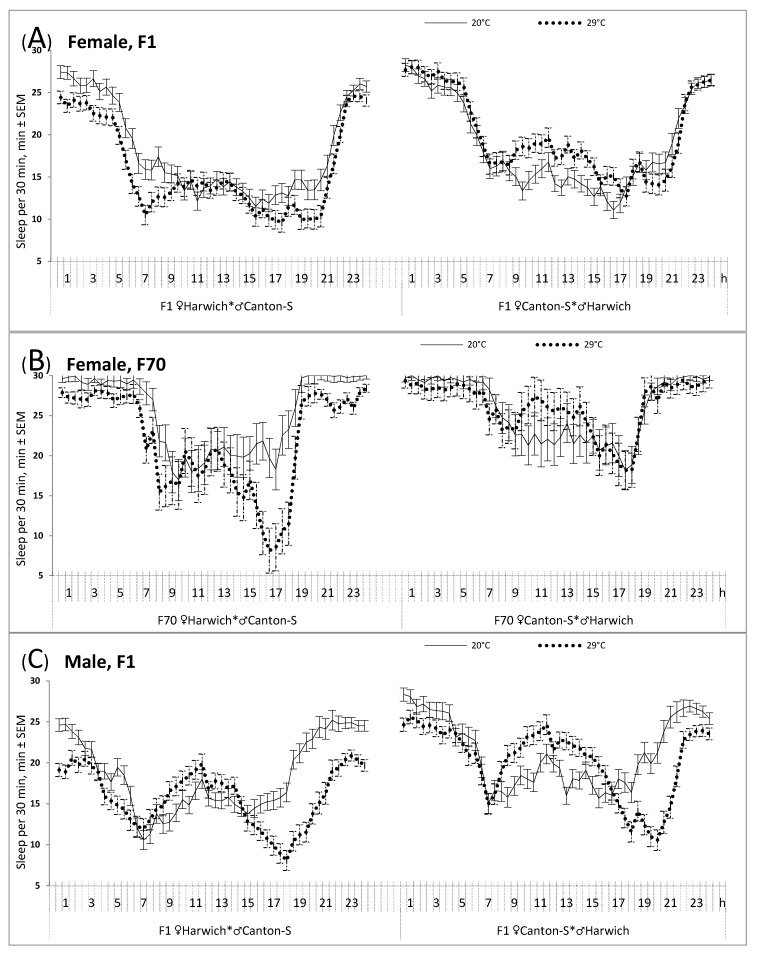
The 24-h sleep patterns in male and female intra-specific hybrids of two cross directions. Sleep, defined [24] as 5 consecutive minutes of absence of any locomotor activity, in min. It was averaged on 30-min intervals; 20 °C and 29 °C: The 24-h pattern in constant darkness under two air temperatures (obtained by averaging over 2nd–5th days and then by averaging over individual flies); (**A**,**B**) Females from F1 and F70, the 1st and 70th generation of hybrids, respectively; (**C**) Males from F1. ♀H♂C-S: Crosses from females of Harwich strain and males of Canton-S strain; ♀C-S♂H: The reciprocal crosses (Canton-S females and Harwich males, females are sterile under high developmental temperature); SEM: Standard Error of Mean; h: Clock Hour. See some results of rANOVAs and sample sizes for each graph in Table 2.

Moreover, the results of the rANOVAs suggested a significant difference between the hybrids of two cross directions in the response of their sleep to high temperature (Figure 2 and Table 2, upper part). For instance, Figure 2A illustrates the significant interaction between independent factors “Cross direction” and “Temperature” yielded by rANOVA of female data (F_1/136_ = 7.357, *p* = 0.008).

Namely, the weakest response was demonstrated by infertile female cross obtained by hybridizing Canton-S females with Harwich males (Figure 2A and Table 2, upper part). Such a difference between female crosses persisted over generations (Figure 2B and Table 2, upper part). It suggested that the infertile (♀C-S♂H) females did not respond to the 29 °C condition by significant decrease of their sleep (Table 2, upper part, and Figure 2A,B, right), whereas a decrease in mean level of sleep in this condition was shown by fertile females (i.e., those born from females of Harwich strain and males of Canton-S strain). In contrast to infertile female hybrids (Figure 2A, right), a rather complex reorganization of the sleep pattern under high temperature was demonstrated by the male hybrids of this cross direction (Figure 2C, right). It included an increase of nighttime activity, a clearer distinguished “siesta”, and an increase of evening activity peak that was shifted to later hours (Figure 2C). The main effect of the independent factor “Temperature” was significant in males of the opposite cross direction (Table 2, upper part, and Figure 2C). They were similar to the female hybrids of this cross direction in showing a decrease in mean level of sleep under high temperature (Table 2).

**Figure 3:**
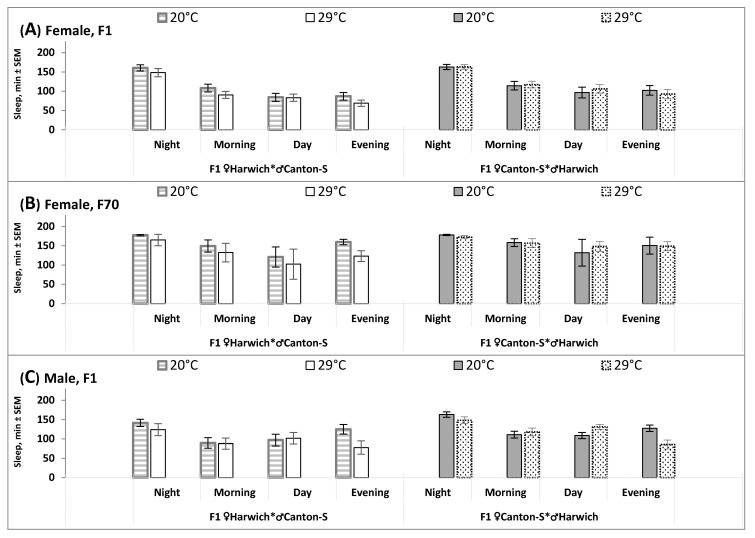
Sleep in four 6-h time intervals of the 24-h cycle in intra-specific hybrids of two cross directions. After averaging sleep over 30-min intervals (Figure 2), it was summed as four 6-h time intervals to compare the amounts of sleep during Night (between 22:00 and 4:00), Morning (between 4:00 and 10:00), Day (between 10:00 and 16:00), and Evening (between 16:00 and 22:00); 20 °C and 29 °C: Two air temperatures; (**A**–**C**) The 1st and 70th generations of female hybrids, respectively (Females, F1 and F70), and the 1st generation of female hybrids (Males, F1). ♀H♂C-S: Crosses from females of Harwich strain and males of Canton-S strain; ♀C-S♂H: The reciprocal crosses (Canton-S females and Harwich males), females are sterile under high developmental temperature (**A**, right) and fertile under normal developmental temperature (**B**, right); SEM: Standard Error of Mean. See the results of t-tests and sample sizes for each plot in Table 3.

An additional analysis applied to sleep data on longer (6-h) intervals of the 24-h cycle (Student’s test) supported these results indicating the influence of cross direction on the response of 24-h sleep pattern to high temperature (Figure 3 and Table 3). Amount of sleep within none of four intervals significantly changed under high temperature condition in the female F1 and F70 obtained by hybridizing Canton-S females with Harwich males (Table 3 and Figure 3A,B, right). In contrast, the most pronounced changes were shown by the males of this cross direction. Their sleep decreased during evening and nighttime, but increase during daytime (Figure 3C, right, and Table 3). Significant changes in amount of sleep in the evening were also found in other hybrids (i.e., the females and males obtained by hybridizing Harwich females with Canton-S males).

Thus, the results on the 24-h sleep pattern indicated that, contrary to our expectations, this pattern was least, not most, affected by high temperature in infertile (♀C-S♂H hybrid) females. Moreover, these females had an enlarged mean level of sleep as suggested by the significant main effect of independent factor “Cross direction” yielded by rANOVA of female data (F_1/136_ = 15.382, *p* < 0.001). Therefore, the exposure to high temperature led to reorganization of the 24-h sleep pattern in the expected way only in fertile crosses (i.e., ♂C-S♀H females and males of any cross direction). In particular, the typical response to this condition was observed in ♀C-S♂H hybrid males (Table 3). The decreased amounts of evening and nighttime sleep were partly compensated by an increase in amount of daytime sleep (“siesta”).

## 4. Discussion

Although recent scientific literature contains reports suggesting a clear connection between sleeping problems and infertility in women, it remains unknown from the results of human studies whether sleep disruptions can have a central deleterious effect on reproduction or whether they are only secondary outcomes of the principle pathological mechanisms underlying this effect [1,9,10]. Therefore, we tried to use *Drosophila melanogaster* as a model for addressing such kinds of research questions with a hope that the experiments with this species can provide possibility to compare sleep disturbances in fertile and infertile females with a very similar genetic background. Namely, this *Drosophila* model of temperature-sensitive sterility allowed the examination of whether the typical temperature-induced disturbances of sleep can be linked to infertility in females of one of two cross directions (females produced by hybridization of females of Canton-S strain with males of Harwich strain, ♀C-S♂H). The expected impact of heat on sleep-wake behavior was tested using data on sleep averaged over 30-min and longer (6-h) intervals of the 24-h cycle. We found that this impact was significant in fertile hybrids (females of the reciprocal cross and males of both crosses), as indicated, for instance, by a statistically significant change in the distribution of sleep over night- and daytime hours (i.e., an appearance of insomnia-like decline of amount of night sleep). Contrary to our expectations, the results also suggested a lower, rather than a higher, responsiveness of sleep of infertile females to high temperature. Unlike three other groups of hybrids, they did not demonstrate such typical for fly and human responses to heat as night sleep disturbance, elongated “siesta”, and delaying shift of evening activity into night hours.

In other words, the present results suggested a rather paradoxical relationship between the effects of high temperature on fertility of hybrid female flies, on the one hand, and on regulation of the 24-h pattern of sleep, on the other hand. In contrast to our expectations, this pattern in sterile F1 female progeny was only slightly disturbed by high temperature, while a more typical response was demonstrated by the fertile hybrids including males of the same cross direction. Namely, the difference of infertile hybrid females from male (always fertile) hybrids was most pronounced because the highest responsiveness to high temperature was showed by males of this and reciprocal cross (i.e., from males of Canton-S strain with females of Harwich strain, ♀H♂C-S). Therefore, the present results failed to provide evidence for a positive correlation between the effects of high temperature on fertility and sleep-wake patterns. On the other hand, the results were positive with respect to the expectation of finding a difference between crosses in the 24-h sleep pattern and finding a difference between them in its responsiveness to high temperature. We documented significant differences between crosses that persisted over generations.

Overall, our results on the 24-h sleep pattern in fertile fruit flies were in full agreement with the findings reported on this pattern in the literature [16,26,27,28,29,30,31]. For instance, males slept more than females during the daytime [32,33], and an increase in ambient temperature caused a decrease of night sleep, an increase of “siesta”, and a shift of the second (evening) activity peak to later (early night) hours [16,17,18,19]. Additionally, we found that the effect of high temperature viewed as both a decrease of the mean level of sleep and an appearance of insomnia-like increase of nighttime activity is not universal. It seems that its strength might vary depending upon cross direction and sex. While this effect was non-significant in infertile female hybrids and rather modest in females of the opposite cross, it was very pronounced in fertile male hybrids, either of this or the opposite cross direction.

The present results also confirmed a remarkable similarity between responses of human’s and fly’s sleep to high temperature. As was shown in the previously reported studies of fruit flies [16,17,18,19], the present study revealed a reorganization of the sleep-wake cycle in response to increase of ambient temperature by a decrease of nighttime sleep, an increase of sleep in the middle of the day, and a decrease of early night sleep. Similar responses have been found in our own species. For instance, as was documented in a study of month-to-month variation in sleeping problems [14,15], more than one-half of native residents and newcomers to a region with hot summer temperatures reported a rise in the prevalence of each of the studied symptoms of sleep disturbance in the summer months (e.g., increase of complaints about daytime sleepiness, difficulties falling and staying asleep, and premature awakening). In the present study of fruit fly, we found that a more promising marker of infertility might be hypersomnia in any of two temperature conditions and an impaired responsiveness of the sleep-wake behavior to stressful levels of temperature. In other words, a predisposition to develop infertility under thermal stress can be linked to a lack of the typical response to heat.

Possibly, the behavior pattern shown by infertile females (females born from females of Canton-S strain and males of Harwich strain, ♀C-S♂H) might be regarded as beneficial because it allows the conservation of energy and lowering overheating caused by muscle activity. Such a suggestion, however, is not fully supported by the results on survival rate in infertile or fertile females of this cross (♀C-S♂H). No difference was found between fertile and infertile F1 or F70 females in duration of lifespan under high temperature. Although energy conservation and overheating avoiding behavior was not translated into a longer lifespan of the infertile females (♀C-S♂H) compared to the fertile female of the opposite cross direction (♀H♂C-S), female hybrids of both crosses lived longer than males and flies of parental strains. Possibly, the contribution of two distant strains into hybrid genome was beneficial for health of females, and it seems that such a hybrid vigor persisted over generations without change in the pattern of response of sleep to high temperature. On the other hand, it is not excluded that infertile hybrid females (♀C-S♂H) can have a poorer health than fertile hybrid females (♀H♂C-S), but they can live for a similar length of time to fertile hybrid females due to an impaired behavioral response to high temperature. Given the complexity of relationship between energy expenditure and longevity [34], additional experiments are required to bring clarity to such results of survival analysis. We can only resume that these intra-specific hybrids of *Drosophila melanogaster* might demonstrate both hybrid vigor and hybrid incompatibility.

The present results on infertility of intra-specific hybrids were in line with the original findings reported by Kidwell et al. [20]. As we expected from this report [20], the symptoms of paternal-maternal hybrid gonadal dysgenesis were identified exclusively in female hybrids from one of two cross directions (i.e., ♀C-S♂H). Since fertility was lost only in the 29 °C condition, we also confirmed that developmental temperature was the critical factor for occurrence of this deleterious effect. Moreover, as we expected from the previous reports on ♀C-S♂H females (e.g., [23,25]), hybrid dysgenesis was found to be primarily associated with germ cell degeneration during the early stages of embryonic development under elevated temperature.

To the best of our knowledge, this was the first attempt to address the question of association between female infertility and sleep disturbances by using such specific animal model as the temperature-dependent infertility of hybrids produced by crossing two distinct strains of *Drosophila melanogaster* followed by an exposure their offspring to high temperature. Since clear signs of such an association were not seen in our experimental study, we did not try to check these particular hybrids on the involvement of physiological, anatomical, genetics, and other possible underlying mechanisms in the week sleep response to high temperature of infertile females. Despite the lack of support for our primary expectation of such an association, additional experimental research of fruit fly’s models of infertility would be helpful for further elaboration of the possible links between the effects of environmental factors on fertility and sleep-wake patterns. For instance, such future study could take into consideration the report by Potdar et al. [35] suggesting that sleep deprivation due to feeding caffeine or due to mechanical perturbation was a cause of decreased egg output, and that transient activation of wake-promoting dopaminergic neurons decreased egg output in addition to decrease in sleep levels. Therefore, one promising direction for future studies using fruit fly models might be to explore the prospects of modeling the effects of acute rather than developmental stressors on fertility and sleep-wake patterns of normally developed flies. Anyway, the present results supported our general suggestion of significant differences between crosses in both sleep pattern and its responsiveness to high temperature. Therefore, such results make it possible to recommend aiming future studies at the experimental exploration of the genetic underpinnings of the mechanisms underlying the response of fruit fly’s sleep to high temperature.

## 5. Conclusions

Given the complexity of the human reproduction system and the numerous limitations imposed on human studies, fruit flies’ model was used to investigate the associations between the effects of high temperature on fertility and 24-h sleep pattern. No evidence was provided for a connection between sleep disturbance and infertility of females born from females of Canton-S strain and males of Harwich strain. However, we found that the 24-h sleep pattern and its responsiveness to high temperature depend upon cross direction. This dependence was observed both in male and female intra-specific hybrids and the difference between female crosses persisted over generations. The 24-h sleep pattern in the infertile female cross was found to be least responsive to high air temperature, and only three other (fertile) crosses demonstrated more or less typical thermal responsiveness. Therefore, female fertility rather than infertility was found to be connected to responsiveness of nighttime sleep to high temperature. Future research of fruit flies’ models of reproductive health might be aimed on exploring the associations of sleep pattern not only with developmental temperature-induced fertility but also with fertility caused by acute deleterious effects of high temperature.

## Figures and Tables

**Table 1:** Survival time, in days, in two strains and their hybrids of two cross directions kept at 29 °C.

Generation				F1				F70	
Sex	Strain	Mean	SEM	−95%	+95%	Mean	SEM	−95%	+95%
Female	♀H♂C-S	34.7 ^a^	0.4	33.9	35.4	34.7	1.0	32.7	36.6
	♂C-S♀H	32.9 ^a,b^	0.6	31.6	34.1	33.5 ^b^	0.8	32.0	35.0
	Harwich	21.2	0.3	20.7	21.8	27.4	0.7	26.1	28.7
	Canton-S	28.8	0.8	27.3	30.3	33.5	0.8	32.0	35.1
Male	♀H♂C-S	33.2	0.3	32.6	33.8	25.3	0.8	23.7	26.9
	♂C-S♀H	32.8	0.5	31.9	33.7	28.1	0.7	26.6	29.5
	Harwich	24.0	0.6	22.8	25.1	20.2	0.7	18.9	21.5
	Canton-S	29.0	0.7	27.6	30.3	27.4	0.9	25.6	29.1

Note. ♀H♂C-S: Crosses of females from Harwich strain with males from Canton-S strain; ♀C-S♂H: Crosses from female Canton-S strain with male Harwich strain (females are infertile under high developmental temperature). F1 and F70: The 1st generation and the 70th generation of intra-specific hybrids; SEM: Standard Error of Mean; −95% and +95%: Confidence interval. According to log rank (Mantel-Cox) test, ^a^: Comparison of females from two crosses (♀H♂C-S vs. ♀C-S♂H in F1) gave *χ*^2^ = 0.004, df = 1, *p* = 0.948, ^b^: Comparison of females from two generations (F1 vs. F70 for ♀C-S♂H) gave *χ*^2^ = 0.003, df = 1, *p* = 0.960. See also results of some other comparisons obtained by using the *χ*^2^ statistic in Results.

**Table 2:** 24-h sleep pattern: effects of independent factors and interactions (between them or with “Time”)

1. Two-Way rANOVAs: Main Effect of “Temperature” and Its Interaction with “Time”						
Sex and F	Female F1 (Figure 2A)		Male F1 (Figure 2C)		Female F70 (Figure 2B)	
Parents	♀H♂C-S	♀C-S♂H	♀H♂C-S	♀C-S♂H	♀H♂C-S	♀C-S♂H
n 20 °C/29 °C	37/38	38/35	37/34	34/32	8/6	8/8
Main effect:	Independent factor «Temperature» (20 °C and 29 °C)					
F-ratio	6.564	1.650	4.686	1.667	3.734	0.046
df	1/69	1/67	1/65	1/60	1/12	1/14
*p*	0.013	0.203	0.034	0.202	0.077	0.834
Interaction:	Between «Time» and «Temperature»					
F-ratio	2.267	3.125	6.232	13.506	2.059	1.266
df	47/3243	47/3149	47/3055	47/2820	47/564	47/658
*p*	0.008	0.001	<0.001	<0.001	0.121	0.299
**2. Four-Way rANOVA of F1: Main Effects and Interactions (Figure 2A,C)**						
Factor	Sex	Cross	Temperature	Sex	Cross	Temperature
Effect:	Main effect of independent factors			Interaction of these factors with “Time”		
F-ratio	1.007	26.825	6.542	19.168	2.357	9.919
df	1/277	1/277	1/277	47/13,019	47/13,019	47/13,019
*p*	0.317	<0.001	0.011	<0.001	0.007	<0.001
Interaction:	Double, between two independent factors			Triple, with “Time”		
Factor	Cross	Temperature	Sex	Cross	Temperature	Sex
F-ratio	0.212	2.738	0.660	3.925	2.059	9.919
df	1/277	1/277	1/277	47/13,019	47/13,019	47/13,019
*p*	0.664	0.099	0.417	<0.001	0.121	<0.001

Note. Sleep, defined as 5 consecutive minutes of absence of any locomotor activity, in min [24], was averaged on 30-min intervals of 24-h cycles on days 2–5. The effect of repeated measure “Time” (48 time points of the 24-h cycle) was always significant (*p* < 0.001). 1. F1 and F70: The 1st and 70th generations of intra-specific hybrids; Cross: Cross direction, either ♀H♂C-S (crosses of females from Harwich strain with males from Canton-S strain) or ♀C-S♂H (crosses of female Canton-S strain with male Harwich strain, F1 females are infertile under high developmental temperature). Factor: Independent Factor; F-ratio, df, and *p*: F-ratio, degree of freedom, and level of significance for either main effect or interaction. Degrees of freedom were corrected using Greenhouse-Geisser correction controlling for type 1 error associated with violation of the sphericity assumption, but the original degrees of freedom are reported in this table and Results. See also some other results of rANOVAs in Results and Figure 2A–C illustrating the interaction of the repeated measure with “Temperature” and “Cross direction”. 2. The remaining interactions (between all three independent factors and their interaction with the repeated measure) were non-significant.

**Table 3:** Amount of sleep in four 6-h time intervals of the 24-h cycle: effect of temperature.

Sex and F	Female F1 (Figure 3A)		Male F1 (Figure 3C)		Female F70 (Figure 3B)	
Parents	♀H♂C-S	♀C-S♂H	♀H♂C-S	♀C-S♂H	♀H♂C-S	♀C-S♂H
n 20 °C/29 °C	37/38	38/35	37/34	34/32	8/6	8/8
*t*-Test:	Night (22:00–04:00), 20 °C vs. 29 °C					
t	−1.827	0.047	−1.914	−2.67	−1.660	−2.601
df	73	71	54.906	64	5.165	8.875
*p*	0.072	0.963	0.061	0.010	0.156	0.029
*t*-Test:	Morning (04:00–10:00), 20 °C vs. 29 °C					
t	−2.595	0.422	−0.159	1.072	−1.233	−0.171
df	73	71	69	64	12	14
*p*	0.011	0.674	0.874	0.288	0.241	0.867
*t*-Test:	Day (10:00–16:00), 20 °C vs. 29 °C					
t	−.186	1.081	0.441	4.339	−0.822	0.878
df	73	71	69	61.614	12	14
*p*	0.853	0.283	0.660	<0.001	0.427	0.395
*t*-Test:	Evening (16:00–22:00), 20 °C vs. 29 °C					
t	−2.861	−1.103	−4.537	−5.948	−4.631	−0.129
df	73	71	69	64	7.519	14
*p*	0.006	0.274	<0.001	<0.001	0.002	0.899

Note. After averaging sleep over 30-min intervals (Figure 2), it was further averaged over four 6-h time intervals (Figure 3) to calculate amount of sleep for Night (between 22:00 and 4:00), Morning (between 4:00 and 10:00), Day (between 10:00 and 16:00), and Evening (between 16:00 and 22:00); 20 °C and 29 °C: Amounts of sleep under two air temperatures were compared. *t*-Test: Student’s test for equality of means under two temperatures; t, df, and *p*: t-statistic, degree of freedom, and level of significance. Degree of freedom for t-test was corrected when the null hypothesis that the variances for male and female are equal was rejected (Levene’s test); Bonferroni adjustment for multiple comparisons (four intervals) gave significant result for *p* < 0.0125 (0.05/4); F1 and F70: The 1st and 70th generation of intra-specific hybrids; Cross: Cross direction, either ♀H♂C-S (crosses of females from Harwich strain with males from Canton-S strain) or ♀C-S♂H (crosses of female Canton-S strain with male Harwich strain, females are infertile under high developmental temperature).

## Data Availability

The data presented in this study are available on request from the corresponding author.
